# The chain mediating role of parenting stress and child maltreatment in the association between maternal adverse childhood experiences and executive functions in preschool children: a longitudinal study

**DOI:** 10.1186/s13034-024-00837-6

**Published:** 2024-11-11

**Authors:** Jinhong Zha, Ruoyu Li, Haiyan He, Peifei Fang, Rongling Huang, Tian Xing, Yuhui Wan

**Affiliations:** 1 College & Hospital of Stomatology, Key Lab. of Oral Diseases Research of Anhui Province, Anhui Medical University, Hefei, China; 2 Department of Maternal, Child & Adolescent Health, School of Public Health, Anhui Medical University, Hefei, Anhui China; 3 Anhui Provincial Key Laboratory of Environment and Population Health across the Life Course, Hefei, Anhui China; 4 Wuhu Maternal and Child Health and Family Planning Service Center, Wuhu, China; 5 Department of Physiology, Anhui Medical College, Hefei, Anhui China; 6 Anhui Women and Children Medical Care Center, Hefei, China

**Keywords:** Maternal adverse childhood experiences, Preschoolers' executive functions, Parenting stress, Child maltreatment, Chain mediating

## Abstract

**Background:**

Previous researches found that maternal adverse childhood experiences not only affect the psychological behavior of preschool children, but also have direct or indirect negative effects on the executive functions and cognition of offspring. And, the possible social psychological mechanism between maternal adverse childhood experiences and preschool children’s executive functions is still not clear.

**Objectives:**

This study mainly tries to understand the association between parenting stress and child maltreatment in maternal adverse childhood experiences and children’s executive functions through longitudinal cohort.

**Participants and setting:**

Participants were 2160 preschool children and their mothers who finally completed baseline and 3 waves of follow-up.

**Methods:**

Using a cohort study, a baseline survey of junior kindergartens was carried out in June 2021 and followed up every six months, with a total of 3 follow-ups.

**Results:**

We found that executive functions in preschoolers were significantly positively correlated with maternal adverse childhood experiences, parenting stress, physical assault, psychological aggression, neglect and nonviolent discipline (*r* = 0.180, 0.386, 0.274, 0.302, 0.189, 0.148, respectively, *P* < 0.01). Further, parenting stress and child maltreatment showed a chain mediating effect between maternal adverse childhood experiences and executive functions in preschoolers, and the total indirect effects accounted for 70.56%, 78.69%, 65.38%, and 68.07% of the total effect, respectively.

**Conclusions:**

This study found that maternal adverse childhood experiences have a significant impact on the executive functions of preschool children, and parenting stress and child maltreatment are the mediating factors of their association, revealing the potential mechanism between the two associations from the perspective of social psychology.

## Introduction

Executive Functions (EFs) refer to higher-level cognitive processes that enable us to plan, direct, monitor, and adjust our behaviors to achieve specific goals. These functions are typically associated with the activity of the prefrontal cortex, particularly the dorsolateral and dorsomedial areas, although they also involve other regions of the brain. EFs encompass, but are not limited to, the following aspects: working memory, inhibitory control, attentional control, planning and organization, flexibility and multitasking [1–3]. A substantial amount of research indicates that well-developed EFs in early childhood lay the foundation for later development in the cognitive, social, and emotional domains. Specifically, children with better EFs showed fewer behavioral problems [4], stronger social skills, and stronger emotional regulation [5, 6]. In addition, EF in preschool children has been shown to predict learning skills and academic achievement, and can serve as a resilient resource for mitigating the negative effects of environmental risks on children’s social adaptation [7, 8]. Previous studies have also shown that preschool Children with abnormal early EF scores have difficulty achieving high scores on general neuropsychological and intelligence tests, and have difficulty successfully completing academic tasks and adapting to various social relationships [9]. There is a rising interest in researching the factors that positively and negatively affect early childhood EFs, especially during the period of their rapid development, given the significance of early EF for preschoolers’ physical and mental development. Family members, particularly parents, and the family environment have the earliest and most significant impact on children’s ability to regulate their behavior, according to bioecological and family systems theories, which hold that the family is the most significant proximate environment for early childhood development [10].

There are many factors that affect EF in preschool children, and the majority of prior studies have concentrated on the relationship between child-level factors and EFs, such as children’s negative emotions [11], temperament [12], trauma exposure and trauma-related symptoms [13], and experiences of abuse [14]. In recent years, some scholars have started to focus on the harmful effects of maternal adverse childhood experiences from the perspective of intergenerational effects, such as how these experiences affect the EF development of their offspring [15, 16]. Adverse childhood experiences, also known as ACEs, are defined as being exposed to otentially traumatic events or environments before the age of 18 years, including any form of abuse (physical, emotional, sexual), neglect (physical neglect, emotional neglect), and family dysfunction (separation or divorce of parents, substance abuse or mental illness in family members) [17]. According to a study on young mothers’ cumulative exposure to ACEs, three or more ACEs were experienced by 55% of the mothers when they were children [18]. Bissonnette was et al. [19] discovered that maternal ACEs were linked to more difficulties in preschool offspring’s general development, including children’s cognitive, motor, language, and social-emotional skills. A study of preschool children aged 17–40 months found that maternal childhood emotional and physical abuse was associated with decreased working memory in their children, but not with decreased inhibitory control and cognitive flexibility [20]. An intergenerational link among maternal childhood emotional and physical abuse and the EFs of preschool children was explored in a 309-pair cohort study of mothers and children in a Chinese cohort. The study assessed the mother’s history of childhood abuse and the EFs of preschool children, including working memory, inhibitory control, and cognitive flexibility, at baseline and follow-up. This study discovered an interaction between maternal childhood emotional abuse and children’s EFs. Specifically, severe childhood emotional abuse experienced by mothers is a direct predictor of a child’s ability to think critically, memory retention, and inhibitory control levels [21]. Thus, it can be seen that maternal ACEs are an important factor affecting the EF development of preschool children.

According to prominent models of child development [22] and bioecological and family systems theories [10], parental factors and the family environment may be important reasons for individual differences in children’s EFs. The results of a meta-analysis suggest that the home environment is particularly important for young children’s EF development [23]. Various factors related to parents, which include socioeconomic position [24], parental education [22], parenting stress [25], parenting style [26], etc., have been demonstrated to be responsible for EFs in children. Alan et al. collected parental stress and parent-reported executive function difficulties of children aged 2 to 12 years (*n* = 243) and found that parent-reported executive function difficulties of children was significantly associated with parenting stress [27]. In addition, a study conducted in China involving 311 preschool-aged children and their parents revealed a notable inverse connection in psychological aggression and corporal punishment and EFs in preschool children [28]. A previous longitudinal study found that negative parenting behaviors had an effect on children’s EFs, while positive parenting behaviors had no effect on children’s EFs [29]. Therefore, parenting stress and child maltreatment are important family environment factors that affect children’s EFs.

In accordance with the theory that biosocial systems, including hereditary factors and socialization experiences, play a role in promoting ideal self-regulated advancement [30], it is expected that parents are anticipated to impact the EFs of preschool children not only through genetic variables but also throughout the environment they establish. At present, in most majority of Chinese families, mothers occupy a major position in the daily companionship and education of their children. In addition, previous studies have clearly shown that maternal ACEs lead to parenting stress [31], child maltreatment [32], and children’s EF development [20], and the above studies have also demonstrated that parenting stress greatly raises the likelihood of child maltreatment, and both parenting stress and child maltreatment are also important factors affecting children’s EFs. Therefore, we speculate that parenting stress and child abuse could be factors that mediate the attachment between maternal ACEs and the EFs of preschool children. Based on the samples of preschool children and their mothers in China, this study hypothesizes that maternal ACEs could directly predict EFs in children, and that parenting stress and child abuse are variables that contribute to the connection between maternal ACEs and children’s EFs, so as to explore the internal mechanism and influencing factors of preschool children’s EFs and provide an important reference for the prevention and intervention of children’s EFs. Research hypotheses for this study:

### Hypothesis 1

Maternal ACEs can significantly predict EFs in preschoolers.

### Hypothesis 2

Maternal ACEs indirectly affect EFs in preschool children by increasing parenting stress.

### Hypothesis 3

Maternal ACEs indirectly affect EFs in preschool children through child maltreatment, comprising physical assault, psychological aggression, neglect, and non-violent discipline.

### Hypothesis 4

Parenting stress and child maltreatment serve as chain mediating role of maternal ACEs with EFs in preschool children.

## Methods

### Participants and procedure

**Fig. 1:**
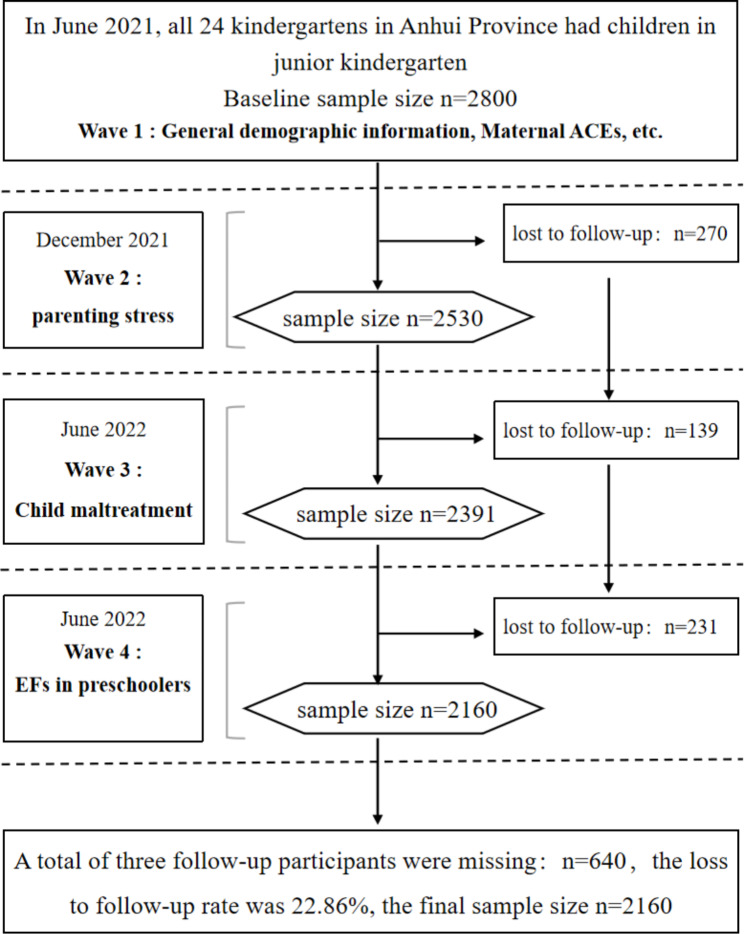
Flowchart of participation for the final analysis sample

This study used data from the Anhui Provincial Preschool Cohort (APCC) study, a school-based longitudinal study that focused on the impact of family environmental factors on the physical and mental health of children aged 3 to 6 years. A total of 24 kindergartens were included, comprising of eight kindergartens selected from each region. Questionnaires were conducted on children and their mothers in kindergarten classes from June 2021 to December 2022. A baseline survey was conducted in June 2021, which was then followed up every six months for a total of three follow-up visits (Fig. 1), with a final loss to follow-up rate of 22.86%. The analysis included data from 2160 children and their mothers.

The survey used online questionnaires to collect information, communicate with the local leaders and kindergarten principals before the survey, and obtain the informed consent of the kindergarten and the children’s parents. Before the survey, a parent-teacher meeting was conducted, in which the enumerator introduced the research purpose of the survey and how to fill in the questionnaire to the parents, and then the mothers of the preschool children filled in the electronic questionnaire. This study has been reviewed by the Ethics Committee of Anhui Medical University (20210655).

### Measurements

#### Maternal ACEs

At the baseline survey (Wave 1), the WHO Adverse Childhood Experiences International Questionnaire (ACE-IQ) was used to evaluate the ACEs of mothers of preschool children before the age of 18 [33]. To ensure the survey was relevant to current Chinese culture, the ACE-IQ was appropriately adjusted in the early stage of the study, such as by deleting the items related to war or gun violence. The final ACE-IQ survey consisted of 21 items in 8 dimensions, including emotional, physical, sexual abuse and emotional, physical neglect, as well as peer bullying, community violence, and family dysfunction. With the exception of family dysfunction, each item in the other dimensions was scored on a 5-point scale ranging from 1 (no) to 5 (always). The family dysfunction component was assessed using a binary scale (1 = yes, 2 = no). In this study, items with results of “occasionally”, “sometimes”, “often”, “always”, or “yes” were defined as positive items and assigned as “1”. Items with a result of “no” were defined as negative items and assigned as “0”. Having one or more positive items in each dimension were defined as having this type of ACE exposure, and the cumulative total ACEs score was 0–8 points. The Chinese version of the ACE questionnaire demonstrates strong reliability and validity [34], with a Cronbach’s alpha coefficient of 0.71.

#### Parenting stress

At the first follow-up survey (Wave 2), the level of parental stress experienced by mothers of preschool children was evaluated using the Parenting Stress Index Short Form (PSI-SF) developed by Abidin [35]. The PSI-SF comprises three dimensions: parenting distress, parent-child dysfunctional interactions, and problematic children, each consisting of 12 items. The questionnaire uses a five-point scoring system for evaluation, from “strongly disagree” (1 point) to “strongly agree” (5 points). The sum of all questions is calculated to calculate the stress score, which correlates with the level of parenting stress. In 1995, Taiwan scholar Ren Wenxiang carried out the localization and revision of PSI-SF, and after testing, the Cronbachα measured by the study was between 0.85 ~ 0.93, indicating that the reliability and validity of the Chinese version of the questionnaire were good. The Cronbach’s alpha coefficient for this study was 0.94. As a short form, PSI-SF is easy to operate, easy to understand and fill in, making it more practical in practical applications, especially suitable for large-scale research and research.

#### Child maltreatment

At the second follow-up survey (Wave 3), child maltreatment was evaluated using the Chinese revised version of the Parent-child Conflict Tactics Scale (CTSPC) developed by Straus et al. [36]. The scale includes a total of 4 subscales: physical assault (13 items), psychological aggression (5 items), neglect (5 items), and non-violent discipline (4 items). Mothers of school-age children were asked to report the number of times they had performed a specific act with their child in the past week: never (0); once (1); a few times (2); many times (3); every time (4). Finally, the scores of the items in each subscale were summed to determine the total score for each dimension. The higher the total score for each dimension, the more severe the mother’s behavior in that domain. A higher total score indicates more pronounced severe behavior by the mother in that domain. The current research found Cronbach’s alpha values of 0.83, 0.76, 0.66, and 0.45 for psychological aggression, physical assault, neglect, and non-violent discipline, respectively. Although the coefficients in this investigation, while not notably high, were in line with findings from other extensive studies utilizing CTSPC [37]. In addition, numerous studies in the Chinese cultural context have proven that the scale is applicable for measuring harsh disciplinary behaviors of parents of preschool children in our country.

#### Executive function

At the third follow-up survey (Wave 4) utilized the Chinese version of the Behavior Rating Scale of Executive Function-Preschool Version (BREIF-P) to assess the EFs of preschool children as reported by their mothers. The BRIEF-P parent version developed by Gioia et al., suitable for children aged 2–5 years and 11 months [38], evaluates children’s EFs in daily life through parents reporting whether there are problems with children’s behavior. The assessment consists of 63 items evaluated on a 3-point Likert-type scale (1 = never a problem, 2 = occasionally a problem, 3 = often a problem). A higher score indicates a more significant impairment of EFs. The questionnaire has a total of five individual subscales and the scores of the five scales are combined into a total executive function (global executive composite, GEC) score. The Cronbach’s alpha value for EF in children was 0.97 in this study. Previous studies have also demonstrated sufficient internal reliability and validity [39, 40], and it has been validated in the Chinese cultural context, making it suitable for children in the Chinese cultural background.

### Demographic characteristics

The study gathered demographic data using a self-created general information questionnaire that covered the child’s age and sex, whether they were born prematurely, if they are a single child, weight at birth, duration of breastfeeding, average daily screen time and daily sleep duration, mother’s age and degree, monthly household income, and family structure.

### Statistical analyses

We used SPSS 23.0 software to conduct data analysis, and Spearman correlation analysis was employed to examine the relationship between maternal ACEs, parenting stress, child maltreatment, and the EFs of preschool children. The research utilized Hayes’s [41] SPSS plug-in PROCESS model 6 to examine how parenting stress and child maltreatment mediate the correlation between maternal ACEs and EF in children, while taking into consideration children’s sex, only child status, and mother’s education level. In this paper, the mediating effect was statistically significant when the 95% CI of the mediating effect size did not include 0. The discrepancy was statistically significant at a level of *P* < 0.05.

## Results

### Description the distribution of general demographic features in both the follow-up and lost-to-follow-up populations

Table 1 shows that there were no significant differences in other general demographic characteristics between the two populations except for the age of the child and whether he was an only child.

**Table 1:** Description of the distribution of general demographic characteristics in the follow-up and lost-to-follow-up populations

Variable	Number	Crowd type		t/χ^2^	*P*
		lost-to-follow-up	follow-up		
Preschooler age	2800	4.14 ± 0.40	4.22 ± 0.34	4.171*	< 0.001
*Preschool child sex*					
Boy	1422	338(52.8%)	1084(50.2%)	1.364	0.243
Girl	1378	302(47.2%)	1076(49.8%)		
*Was the child born prematurely*					
Premature birth	120	26(4.1%)	94(4.4%)	0.101	0.751
Not premature	2680	614(95.9%)	2066(95.6%)		
*Single child*					
Yes	1030	206(32.2%)	824(38.1%)	7.543	0.006
No	1770	434(67.8%)	1336(61.9%)		
*Weight at birth*					
<2.5 kg	105	21(3.3%)	84(3.9%)	1.093	0.579
2.5 ~ 4kg	2273	516(80.6%)	1757(81.3%)		
≥ 4 kg	422	103(16.1%)	319(14.8%)		
*Duration of breastfeeding*					
≤ 6 months	838	205(32.0%)	633(29.3%)	1.927	0.382
6 months ~ 1 year	1338	293(45.8%)	1045(48.4%)		
>1 year	624	142(22.2%)	482(22.3%)		
*Average daily screen time*					
No	143	41(6.4%)	102(4.7%)	4.458	0.216
≤ 1 h	1395	305(47.7%)	1090(50.5%)		
1 ~ 2 hours	1035	236(36.9%)	799(37.0%)		
>2 h	227	58(9.1%)	169(7.8%)		
*Average daily sleep duration*					
≤ 10 h	1294	304(47.5%)	990(45.8%)	1.596	0.450
10 ~ 13 hours	1491	331(51.7%)	1160(53.7%)		
>13 h	15	5(0.8%)	10(0.5%)		
Mother’s age	2800	33.29 ± 4.92	32.92 ± 4.43	−1.719*	0.086
Maternal ACEs	2800	2.47 ± 1.86	2.22 ± 1.66	−3.082*	0.002
Parenting stress	2530	80.29 ± 19.02	79.07 ± 19.48	−1.093*	0.274
*Mother’s degree*					
Junior high school and below	596	133(20.8%)	463(21.4%)	1.168	0.558
High school, technical secondary school	728	158(24.7%)	570(26.4%)		
College degree or above	1476	349(54.5%)	1127(52.2%)		
*Monthly household income*					
≤ 3000 RMB	252	51(8.0%)	201(9.3%)	3.624	0.305
3000 ~ 6000 RMB	1074	244(38.1%)	830(38.4%)		
6000 ~ 10000 RMB	923	204(31.9%)	719(33.3%)		
>10,000 RMB	551	141(22.0%)	410(19.0%)		
*Family structure*					
Extended family	1229	282(44.1%)	947(43.8%)	0.107	0.948
Nuclear family	1505	344(53.8%)	1161(53.8%)		
Other families	66	14(2.2%)	52(2.4%)		

*t, the remainder is *χ*^2^

### Conducting correlation analysis on all variables

Table 2 depicts the data from the statistical analyses and correlation analysis of maternal ACEs, parenting stress, child maltreatment and EFs in preschoolers. Correlation analysis showed that EF in preschoolers (89.79 ± 18.64) was significantly positively correlated with maternal ACEs (*r* = 0.180, *P* < 0.01), parenting stress (*r* = 0.386, *P* < 0.01), physical assault (*r* = 0.274, *P* < 0.01), psychological aggression (*r* = 0.302, *P* < 0.01), neglect (*r* = 0.189, *P* < 0.01), and non-violent discipline (*r* = 0.148, *P* < 0.01). Maternal ACEs (2.22 ± 1.66) were positively correlated with parenting stress (*r* = 0.280, *P* < 0.01), physical assault (*r* = 0.238, *P* < 0.01), psychological aggression (*r* = 0.263, *P* < 0.01), neglect (*r* = 0.155, *P* < 0.01), and non-violent discipline (*r* = 0.128, *P* < 0.01). Parenting stress (79.07 ± 19.48) was positively correlated with physical assault (*r* = 0.284, *P* < 0.01), psychological aggression (*r* = 0.327, *P* < 0.01), neglect (*r* = 0.248, *P* < 0.01), and non-violent discipline (*r* = 0.057, *P* < 0.01). Notably, there was a significant correlation between all variables, which could be used to test the mediating effect.

**Table 2:** Descriptive statistics and correlation analysis between maternal ACEs, parenting stress, child maltreatment and executive function in preschoolers

	$\:\stackrel{-}{\:x}\:$± s	1	2	3	4	5	6	7
1. Maternal ACEs	2.22 ± 1.66	1.000						
2. Parenting stress	79.07 ± 19.48	0.280**	1.000					
3. Physical assault	7.15 ± 3.07	0.238**	0.284**	1.000				
4. Psychological aggression	4.70 ± 3.47	0.264**	0.327**	0.612**	1.000			
5. Neglect	2.45 ± 3.87	0.155**	0.248**	0.248**	0.281**	1.000		
6. Non-violent discipline	0.75 ± 1.48	0.128**	0.057**	0.266**	0.393**	0.067**	1.000	
7. Executive function	89.79 ± 18.64	0.180**	0.386**	0.274**	0.302**	0.189**	0.148**	1.000

*N* = 2160, **P < 0.01

### Mediation analysis

When parenting stress and child maltreatment (physical assault, psychological aggression, neglect, and non-violent discipline) are used as mediating variables, results showed (Table 3) that maternal ACEs significantly positively predicted parenting stress (*β* = 3.244, *P* < 0.001), physical assault (*β* = 0.288, *P* < 0.001), EFs in preschoolers (*β* = 0.552, *P* < 0.05); psychological aggression (*β* = 0.355, *P* < 0.001); neglect (*β* = 0.076, *P* < 0.001), EFs in preschoolers (*β* = 0.649, *P* < 0.01); non-violent discipline (*β* = 0.207, *P* < 0.001), EFs in preschoolers (*β* = 0.599, *P* < 0.05). Parenting stress significantly and positively predicted physical assault (*β* = 0.051, *P* < 0.001), EFs in preschoolers (*β* = 0.322, *P* < 0.001); psychological aggression (*β* = 0.053, *P* < 0.001), EFs in preschoolers (*β* = 0.304, *P* < 0.001); neglect (*β* = 0.015, *P* < 0.001), EFs in preschoolers (*β* = 0.338, *P* < 0.001); non-violent discipline (*β* = 0.008, *P* < 0.05), EFs in preschoolers (*β* = 0.348, *P* < 0.001). Child maltreatment (physical assault, psychological aggression, neglect, and non-violent discipline) significantly positively predicted EFs in preschoolers (*β* = 0.616, *P* < 0.001; *β* = 0.929, *P* < 0.001; *β* = 1.051, *P* < 0.001; *β* = 0.630, *P* < 0.001). With the addition of parenting stress and child maltreatment as mediating variables, the positive prediction of maternal ACEs on the EFs in preschoolers remained significant (*β* = 1.875, *P* < 0.001).

**Table 3:** Multivariate regression analysis results among maternal ACEs, parenting stress, child maltreatment, and executive functions in preschool children(*n* = 2160)

Regression equations			Overall fit factor			Regression coefficient and significance	
Child maltreatment mediating variable	Outcome variable	Predictive variable	R	R^2^	F	*β*	t
Physical assault							
	Parenting stress		0.383	0.146	73.826		
		Gender of the child				−1.749	−2.253*
		Single child				−2.380	−2.868**
		Mother’s degree				−4.723	−9.313***
		Monthly household income				−1.708	−3.855***
		Maternal ACEs				3.244	13.893***
	Physical assault		0.328	0.108	43.253		
		Gender of the child				−0.715	−4.523***
		Single child				0.283	1.671
		Mother’s degree				−0.006	−0.056
		Monthly household income				0.085	0.938
		Maternal ACEs				0.288	5.803***
		Parenting stress				0.051	11.588***
	Executive function		0.415	0.172	64.018		
		Gender of the child				−0.838	−1.139
		Single child				2.627	3.350***
		Mother’s degree				−0.374	−0.768
		Monthly household income				0.033	0.079
		Maternal ACEs				0.552	2.385*
		Parenting stress				0.322	15.385***
		Physical assault				0.616	6.163***
	Executive function		0.203	0.041	18.575		
		Gender of the child				−1.896	−2.410*
		Single child				1.960	2.329*
		Mother’s degree				−2.046	−3.980***
		Monthly household income				−0.518	−1.153
		Maternal ACEs				1.875	7.921***
Psychological aggression							
	Psychological aggression		0.395	0.156	66.257		
		Gender of the child				−0.290	−2.103*
		Single child				0.487	3.299***
		Mother’s degree				−0.190	−2.070*
		Monthly household income				0.052	0.659
		Maternal ACEs				0.355	8.204***
		Parenting stress				0.053	13.942***
	Executive function		0.428	0.183	68.887		
		Gender of the child				−1.009	−1.385
		Single child				2.349	3.009**
		Mother’s degree				−0.201	−0.415
		Monthly household income				0.037	0.090
		Maternal ACEs				0.400	1.724
		Parenting stress				0.304	14.420***
		Psychological aggression				0.929	8.167***
Neglect							
	Neglect		0.283	0.080	31.220		
		Gender of the child				−0.045	−0.741
		Single child				−0.104	−1.589
		Mother’s degree				−0.163	−4.000***
		Monthly household income				−0.051	−1.466
		Maternal ACEs				0.076	3.955***
		Parenting stress				0.015	8.658***
	Executive function		0.405	0.164	60.375		
		Gender of the child				−1.231	−1.672
		Single child				2.911	3.693***
		Mother’s degree				−0.206	−0.419
		Monthly household income				0.140	0.331
		Maternal ACEs				0.649	2.802**
		Parenting stress				0.338	16.273***
		Neglect				1.051	4.063***
Non-violent discipline							
	Non-violent discipline		0.217	0.047	17.760		
		Gender of the child				−0.234	−1.804
		Single child				0.424	3.060**
		Mother’s degree				0.569	6.600***
		Monthly household income				0.066	0.892
		Maternal ACEs				0.207	5.095***
		Parenting stress				0.008	2.140*
	Executive function		0.410	0.168	62.091		
		Gender of the child				−1.131	−1.539
		Single child				2.534	3.217**
		Mother’s degree				−0.737	−1.492
		Monthly household income				0.044	0.104
		Maternal ACEs				0.599	2.584*
		Parenting stress				0.348	17.096***
		Non-violent discipline				0.630	5.161***

*P < 0.05, **P < 0.01, ***P < 0.001.

**Fig. 2:**
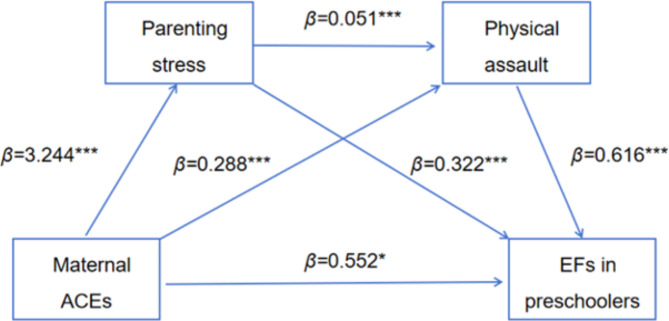
The chain mediation model for maternal ACEs, parenting stress, physical assault, and EFs in preschoolers. ***P < 0.001, **P<0.01, *P<0.05

**Fig. 3:**
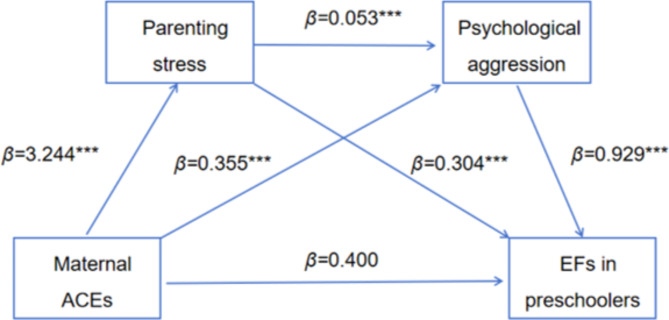
The chain mediation model for maternal ACEs, parenting stress psychological aggression and EFs in preschoolers. ***P < 0.001, **P<0.01, *P<0.05

**Fig. 4:**
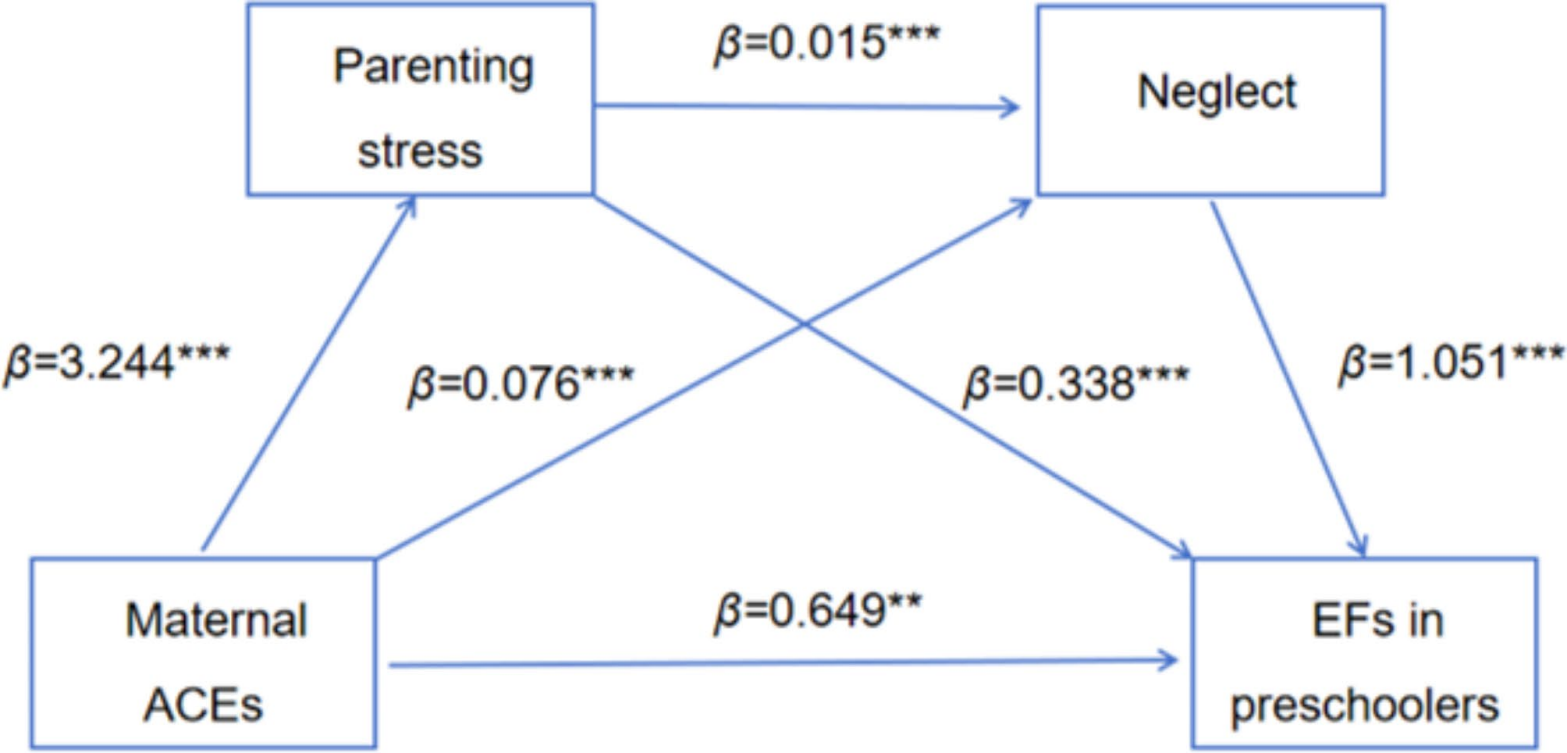
The chain mediation model for maternal ACEs, parenting stress neglect, and EFs in preschoolers. ***P < 0.001, **P<0.01, *P<0.05

**Fig. 5:**
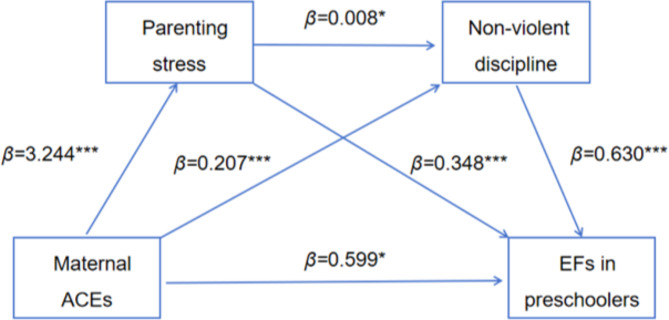
The chain mediation model for maternal ACEs, parenting stress. non-violent discipline and EFs in preschoolers. ***P < 0.001, **P<0.01, *P<0.05

Table 4 shows the chain mediating effects of parenting stress and child maltreatment (physical assault, psychological aggression, neglect, and non-violent discipline) on the relationship between maternal ACEs and EFs in preschoolers. (1) When parenting stress and physical aggression are intermediate variables (Fig. 2), the indirect effect of the single path with parenting stress or physical aggression as the mediating variable were 1.045 and 0.177 (95% CI = [0.849, 1.265], 95% CI = [0.092, 0.283]), respectively; the indirect effects of the chain path with parenting stress and physical aggression as the mediating variable were 0.101 (95% CI = [0.058, 0.158]); the total of all indirect effects was 1.323 (95% CI = [1.093, 1.588]); the effects of the three indirect paths and the total indirect path accounted for 55.71%, 9.44%, 5.41% and 70.56% of the total, respectively. (2) When parenting stress and psychological aggression are intermediate variables (Fig. 3), the indirect effect of the single path with parenting stress or psychological aggression as the mediating variable were 0.986 and 0.330 (95% CI = [0.792, 1.199], 95% CI = [0.222, 0.451]), respectively; the indirect effects of the chain path with parenting stress and psychological aggression as the mediating variable were 0.161 (95% CI = [0.110, 0.220]); the total of all indirect effects was 1.476(95% CI = [1.242, 1.724]); the effects of the three indirect paths and the total indirect path accounted for 52.55%, 17.57%, 8.57% and 78.69% of the total, respectively. (3) When parenting stress and neglect are intermediate variables (Fig. 4), the indirect effect of the single path with parenting stress or neglect as the mediating variable were 1.096 and 0.080 (95% CI = [0.903, 1.325], 95%CI = [0.028, 0.154]), respectively; the indirect effects of the chain path with parenting stress and neglect as the mediating variable were 0.050 (95% CI = [0.020, 0.093]); the total of all indirect effects was 1.226 (95% CI = [1.006, 1.480]); the effects of the three indirect paths and the total indirect path accounted for 58.44%, 4.27%, 2.67% and 65.38% of the total, respectively. (4) When parenting stress and non-violent discipline are intermediate variables (Fig. 5), the indirect effect of the single path with parenting stress or non-violent discipline as the mediating variable were 1.131 and 0.131 (95% CI = [0.915, 1.351], 95% CI = [0.068, 0.208]), respectively; the indirect effects of the chain path with parenting stress and non-violent discipline as the mediating variable were 0.016 (95% CI = [0.002, 0.035]); the total of all indirect effects was 1.277 (95% CI = [1.047, 1.508]); the effects of the three indirect paths and the total indirect path accounted for 60.28%, 6.96%, 0.84% and 68.07% of the total, respectively.

**Table 4:** Chain mediating effects of maternal ACEs, parenting stress, child maltreatment and executive function in preschoolers (*n* = 2160)

Child maltreatment mediating variable	Path	Effect	Boot SE	Percentage of effect size	95% CI	
					BootLLCI	BootULCI
Physical assault	Total effect	1.876	0.237		1.411	2.340
	Direct effect	0.552	0.232	29.44%	0.098	1.006
	Maternal ACEs→parenting stress→EFs	1.045	0.104	55.71%	0.849	1.265
	Maternal ACEs→physical assault→EFs	0.177	0.049	9.44%	0.092	0.283
	Maternal ACEs→parenting stress→physical assault→EFs	0.101	0.026	5.41%	0.058	0.158
	Total indirect effect	1.323	0.126	70.56%	1.093	1.588
Psychological aggression	Total effect	1.876	0.237		1.411	2.340
	Direct effect	0.400	0.232	21.31%	−0.055	0.854
	Maternal ACEs→parenting stress→EFs	0.986	0.104	52.55%	0.792	1.199
	Maternal ACEs→psychological aggression→EFs	0.330	0.059	17.57%	0.222	0.451
	Maternal ACEs→parenting stress→psychological aggression→EFs	0.161	0.028	8.57%	0.110	0.220
	Total indirect effect	1.476	0.126	78.69%	1.242	1.724
Neglect	Total effect	1.876	0.237		1.411	2.340
	Direct effect	0.649	0.232	34.62%	0.195	1.104
	Maternal ACEs→parenting stress→EFs	1.096	0.107	58.44%	0.903	1.325
	Maternal ACEs→neglect→EFs	0.080	0.032	4.27%	0.028	0.154
	Maternal ACEs→parenting stress→neglect→EFs	0.050	0.019	2.67%	0.020	0.093
	Total indirect effect	1.226	0.120	65.38%	1.006	1.480
Non-violent discipline	Total effect	1.876	0.237		1.411	2.340
	Direct effect	0.599	0.232	31.93%	0.144	1.053
	Maternal ACEs→parenting stress→EFs	1.131	0.110	60.28%	0.915	1.351
	Maternal ACEs→non-violent discipline→EFs	0.131	0.036	6.96%	0.068	0.208
	Maternal ACEs→parenting stress→non-violent discipline→EFs	0.016	0.009	0.84%	0.002	0.035
	Total indirect effect	1.277	0.117	68.07%	1.047	1.508

## Discussion

This study developed a chain mediation model to investigate how maternal ACEs, parenting stress, child maltreatment, and EFs in preschool children are interconnected. This study has shown that maternal ACEs have direct and indirect effects on EFs in preschool children, suggesting that maternal ACEs are a key factor affecting EFs in preschool children, validating Hypothesis 1. This aligns with the conclusions of previous researchers. A study of preschool children aged 17–40 months found that maternal childhood emotional and physical abuse was associated with decreased working memory in their children, but not with decreased inhibitory control and cognitive flexibility [20]. Bissonnette was et al. [19] discovered that maternal ACEs were linked to more difficulties in preschool offspring’s general development, including children’s cognitive, motor, language, and social-emotional skills.

Furthermore, this study shows that maternal ACEs can affect the EFs of preschool children through parenting stress, validating Hypothesis 2. Similarly, previous studies have suggested that maternal ACEs are an important risk factor for later parenting stress and can significantly predict later parenting stress (e.g., at preschool age) [42, 43]. Brittany et al. [26] used the New Haven Mental health Outreach for MotherS (MOMS) Partnership to find that mothers experiencing more ACEs was positively correlated with mothers’ current parenting stress, and that this was a dose-response association. High levels of parenting stress led to decreased maternal sensitivity [44] and an increase in various health problems in early childhood, such as emotional-behavioral problems and executive dysfunction [27, 45].

In addition, Kistin et al. [46] used one-on-one qualitative interviews to find that low-income mothers with a history of trauma tend to use harsh or punitive discipline methods, such as establishing discipline as a means of controlling their children and separating from them for long periods of time as a form of punishment. A systematic review also demonstrated a clear link between maternal ACEs and improper parenting of children [47]. A growing body of research indicates that such forms of physical abuse and neglect have a negative effect on children’s EFs. As an example, negative parental control and power assertive strategies can hinder a child’s internalization and self-regulation [48], and negative parent-child interactions, including negative control, negative affect, and corporal punishment, can also adversely affect children’s EFs [49, 50]. Combined with the influence of maternal ACEs on parenting behaviors, it is reasonable to speculate that the association between maternal ACEs mediated by psychological aggression and corporal punishment is consistent with Hypothesis 3 of this study.

Moreover, parenting stress and child abuse act as intermediary factors between maternal ACEs and EFs in preschoolers. This study indicates that the more serious the maternal ACEs are, the greater the impact on her own parenting stress in adulthood, which leads to higher levels of maltreatment and neglect, and increasing EF problems. This is consistent with previous studies showing that mothers who have encountered ACEs are more prone to experiencing elevated levels of parenting stress and engaging in child maltreatment compared to those who have not experienced ACEs [31, 43]. In earlier research, some scholars have focused on the high level of parenting pressure of parents to elevate the likelihood of child abuse. A study conducted through face-to-face interviews in the United States gathered data from Asian American parents regarding their parenting stress and risk of child maltreatment, the findings indicated a notable positive correlation between parenting stress and the likelihood of child maltreatment, even when considering other influencing factors [51]. Crouch et al. discovered that parents with high levels of parenting stress were three times more likely to maltreat their children compared to parents with moderate levels of parenting stress [32]. Obviously, elevated levels of parenting stress can heighten the likelihood of child abuse, both of which affect children’s cognitive development, such as EFs [22, 52]. For example, in a previous study, there was a significant correlation between parent-reported parental stress and children’s EFs between the ages of 2 and 12 years [27]. A study comparing executive functions in children who suffered neglect only against those who experienced neglect plus physical abuse revealed that children who received neglect and physical abuse had poorer scores in problem-solving and planning compared to those who experienced neglect alone [53]. Previous studies have tried to explore the possible mechanisms from a biological perspective. Studies have found that maternal ACEs may alter the cerebral cortex circuits of offspring through the intrauterine environment and HPA axis function during pregnancy [54, 55], thereby affecting volitional activity, emotion regulation, and cognitive function. EFs are higher-level cognitive functions which direct behavior used to achieve all goals, and consist of seemingly unrelated but interrelated functions such as inhibition, switching, working memory, and cognitive flexibility [1, 2]. The prefrontal cortex controls EFs. Thus, maternal ACEs can affect the efficiency and function of the prefrontal cortex and affect the EFs of children [19]. In addition, a survey of 634 Chinese preschool children and their parents revealed that high parenting stress increased the passing down of psychological aggression and physical assault between generations, whereas low parenting stress reduced the transmission of psychological aggression and even hindered the transmission of physical assault. Simultaneously, the study highlighted that parenting stress had a more pronounced moderating effect on transmission in mothers compared to fathers, underscoring the significance of proximal environmental factors, such as parental stress, on the influence of severe discipline [56]. In light of the above discussion, it can be speculated that maternal ACEs may indirectly affect EFs in preschool children through parental stress and child maltreatment, which is the fourth hypothesis of this study.

At the same time, the research findings indicate that although parenting stress and various forms of child maltreatment (including physical assault, psychological aggression, neglect, and non-violent discipline) both have significant impacts on children’s EFs, they differ in the strength and direction of their mediating effects. Parenting stress plays a stronger mediating role between maternal ACEs and children’s EFs, while the mediating effect of physical assault is relatively weaker. This may suggest that parenting stress is a key pathway linking mothers’ experiences with developmental issues in children. Among all the types of maltreatment analyzed, psychological aggression has the strongest mediating effect, emphasizing the profound impact it may have on children’s development. Furthermore, although the mediating effect of neglect exists, it is relatively small, which may reflect the indirect influence of neglectful behavior on children’s EFs. Among the four serial mediating pathways, non-violent discipline has the weakest mediating effect, suggesting that compared to other forms of maltreatment, its negative impact on children’s EFs is relatively minor. The study results indicate that there are differences in the magnitude of mediating effects between parenting stress and different forms of child maltreatment in the relationship between maternal ACEs and children’s EFs. This emphasizes the importance of considering the significance of different forms of maltreatment in future prevention and intervention strategies, with a particular focus on physical and psychological aspects. It is also recommended that future research should further explore these different dimensions of mediating effects to more effectively propose targeted intervention measures.

### Limitations

This study is a cohort study, which explored the intergenerational effect of maternal ACEs by collecting four waves of follow-up data and revealed the connection between maternal ACEs and the EFs of preschool children. However, the study has certain limitations that necessitate further refinement. Specifically, the study did not take into account the baseline executive functions of the children during the design and analysis phases, a factor that directly influences subsequent parenting stress and child maltreatment. Consequently, accurate causal inferences based on the chronological order cannot be made, representing one of the study’s limitations. Additionally, there are other limitations that require improvement. Firstly, there is some recall bias and information bias when assessing maternal ACEs only using retrospective and self-reports, and future studies will add additional assessment of the maternal ACEs, such as standardized diagnostic assessment or physiological measures (such as startle response) to confirm our intergenerational findings. Secondly, the study subjects came from three cities in Anhui Province, and there were differences in the characteristics of the lost and non-lost populations, including the preschooler age, whether they were an only child, and the mothers’ scores on ACEs. Furthermore, the study had a high rate of loss to follow-up, and no measures were taken to address the potential bias associated with the attrition, which limits the generalizability of the conclusions. Future research should increase the sample size and diversity of the population, and similar studies involving larger sample sizes in all regions of the country could be explored. Thirdly, the study collected data during and after the pandemic, and COVID-19 related measures such as stay-at-home orders and social distancing, as well as economic uncertainty, reduced family income, closures of workplaces and schools, and health concerns due to the pandemic, may have led to higher stress levels in mothers and potentially increased the incidence of child maltreatment. This indirectly affects our research results. Therefore, new cohort studies are needed later to verify the results. Fourthly, due to the limitations in data collection and constraints in study design, we were unable to control for confounding factors such as the clustering effect of kindergartens and the mental health of mothers and children. This may result in the heterogeneity of kindergarten environments and resources, as well as mental health issues, affecting children’s development and behavior, thereby influencing our research outcomes to some extent. Future research will consider collecting more data on these relevant confounding factors to better ensure the reliability of the study results. Fifthly, in this study, all variables are derived from maternal reports, and especially when reports of child abuse experiences rely on information provided by potential perpetrators, there may be issues of underreporting or bias, which introduces some limitations to the conclusions. In the future, we will make every effort to collect data from multiple sources, including but not limited to children, other family members, educators, healthcare professionals, and social service records. This multi-source data approach helps to provide a more comprehensive assessment of abuse situations, thereby reducing bias. Sixthly, this study only considers the intergenerational effects of adverse childhood experiences and parenting stress on children’s executive functions from the mother’s perspective, lacking consideration of fathers or other caregivers, which may lead to biases in the research results. Future in-depth research should include data on fathers and other caregivers to provide a more comprehensive perspective. Seventhly, in this study, the variable of child maltreatment was explored in four dimensions separately, and the results of different dimensions showed a certain degree of similarity. However, due to the limitations of sample size and data structure, traditional multiple comparison adjustments could not be performed, which imposes certain limitations on the clear articulation of research conclusions. Therefore, in future research directions, larger sample sizes and more complex statistical models should be used to better control for multiple comparison issues. Finally, the study focused on the mediating role of parenting stress and child maltreatment in the connection between maternal ACEs and preschool children’s EFs, and did not take into account other factors that may affect children’s EFs, such as socioeconomic status, family atmosphere, etc., those factors should be investigated in future follow-up studies.

## Conclusion

A current longitudinal study in Anhui Province examined preschool children and their mothers in three regions, finding a connection between maternal ACEs and the EFs development of preschool children. Furthermore, the connection between maternal ACEs and the EFs of preschool children is mainly mediated by parenting stress and child maltreatment. These findings suggest that in the future, we should actively pay attention to maternal ACEs and parenting pressure, and provide targeted mitigation measures to minimize intergenerational transmission, which is important for protection and prevention during the physical and mental health development of preschool children.

## Data Availability

No datasets were generated or analysed during the current study.
